# A Novel Strategy for Detecting Permittivity and Loss Tangent of Low-Loss Materials Based on Cylindrical Resonant Cavity

**DOI:** 10.3390/s23125469

**Published:** 2023-06-09

**Authors:** Jin Zou, Chuan-Jian Li, Chen Zheng, Dong Wang, Jian Zhang, Xin Wang, Jun-Ying Zhang, Zhi-Ling Hou

**Affiliations:** 1 College of Mathematics and Physics & Being Key Laboratory of Environmentally Harmful Chemical Analysis, Beijing University of Chemical Technology, Beijing 100029, China; 2 Aerospace Institute of Advanced Materials & Processing Technology, Beijing 100074, China; zhengchen2603@163.com (C.Z.); firebatgo@163.com (J.Z.); 3 Second Military Representative Office of Air Force, Beijing 100074, China; idwd@163.com; 4 Beijing Research Institute of High-Tech, Beijing 100094, China; wangxinwx9876@163.com

**Keywords:** low-loss materials, permittivity, loss tangent, cylindrical resonant cavity

## Abstract

Accurate measurement of the permittivity and loss tangent of low-loss materials is essential due to their special applications in the field of ultra large scale integrated circuits and microwave devices. In this study, we developed a novel strategy that can accurately detect the permittivity and loss tangent of low-loss materials based on a cylindrical resonant cavity supporting the *TE*_111_ mode in X band (8–12 GHz). Based on an electromagnetic field simulation calculation of the cylindrical resonator, permittivity is precisely retrieved by exploring and analyzing the perturbation of the coupling hole and sample size on the cutoff wavenumber. A more precise approach to measuring the loss tangent of samples with various thicknesses has been proposed. The test results of the standard samples verify that this method can accurately measure the dielectric properties of samples that have smaller sizes than the high Q cylindrical cavity method.

## 1. Introduction

With the rapid development of 5G technologies and smart sensors [1,2], low-loss dielectric materials have become a hot research topic in various fields, including applications [3,4,5] in the field of ultra large scale integrated circuits [5,6,7], microwave devices, and aerospace [8,9,10,11]. It is essential to accurately test the permittivity and loss tangent of low-loss dielectric materials to meet the application requirements of various fields. Generally, the permittivity and loss tangent of low-loss dielectric materials are mainly measured by the resonant cavity method, including microstrip resonant cavities [12,13,14], rectangular resonant cavities [15,16,17,18], cylindrical resonant cavities [19,20,21], and re-entrant cavity resonators [22]. For example, Federico Gabriele et al. [23] provided relative permittivity measurements for emerging 5G and beyond applications operating at high frequencies based on resonant cavities with the substrate-integrated waveguide technique. Considering low-cost, easy to fabricate, non-contact microwave sensors, Javed Ahmed et al. [24] proposed a cost-effective and convenient solution for accurate permittivity characterization of liquids and binary aqueous solutions with less than 5% percentage error between the calculated and reference permittivity. Karami Mahmood et al. [16] proposed an improved rectangular resonant cavity consisting of a rectangular waveguide and two frequency selective coupling end plates, which enhanced the output power at the resonant frequency of the cavity and improved the determinism of the imaginary part of the permittivity by about 184%. Kopyt Pawel et al. [25] used a combination of several split-column dielectric resonators and a cylindrical cavity (supporting multiple *TM*_0n0_ modes in the microwave band) to measure the permittivity and loss tangent of selected isotropic and anisotropic laminar materials, proposing a more accurate method to measure the out-of-plane component of the complex permittivity of laminar samples. Of course, the most accurate method for measuring electromagnetic parameters of low-loss dielectric materials is the *TE*_011_ mode cylindrical cavity (high Q) method [26,27,28] due to its higher Q value compared to other resonant modes. Generally, the high Q cavity method requires a relatively large sample diameter. If the diameter of the sample does not meet the requirements of the high Q cavity, the resonant modes inside the cavity will be inevitably affected, leading to inaccurate characterization of the dielectric parameters. Moreover, to measure dielectric parameters by current cylindrical cavity methods, it is necessary to know the range of permittivity of the material to be tested in advance. Before the resonant cavity method is used for testing, a sample with a specific thickness needs to be prepared according to the permittivity, and then it is hard to accurately measure the dielectric parameters for materials with unknown permittivity. Therefore, it is of great importance to develop a novel strategy for accurately detecting the permittivity and loss tangent of low-loss materials with smaller samples.

In this paper, a novel technique for measuring complex permittivity using a *TE*_111_-mode cylindrical resonator is proposed by introducing the perturbation of the cutoff wavenumber caused by the coupling hole and sample size, which can be used for accurate retrieval of the permittivity and loss tangent without knowing the permittivity in advance. At the same time, it can accommodate small-sized samples with a diameter of about 29 mm, which is much smaller than that of the high Q cavity method.

## 2. Design and Methods

A cylindrical resonant cavity can be understood as a closed cavity formed by a circular waveguide with a bottom diameter of *D = 2a* on the surface above and below. According to the basic law of an electromagnetic field, electromagnetic waves are totally reflected in an electric ideal wall (short metal surface), thus resonating in the empty waveguide between two electric walls and forming a standing wave. Furthermore, the electric field strength is zero at the port, forming *p* half-wavelength resonant cavity, and the electric field transverse component and the magnetic field transverse component have a phase difference of π/2. The electromagnetic field distribution in the cylindrical resonant cavity can be obtained from the basic solutions of the circular waveguide plus the short-circuit boundary condition of the field distribution at both ends. The mode *TE*_mn_ in the circular waveguide plus the boundary condition in the *z*-direction correspond to the *TE*_mnp_ mode in the cylindrical resonant cavity, where *m*, *n,* and *p* represent the number of maximum values of *E* in φ, *r,* and *z* directions in cylindrical coordinates, respectively.

After mastering the electromagnetic field distribution law inside the resonant cavity, the size of the resonant cavity is reasonably designed according to the actual demands and processing conditions of the samples. To purify the *TE*_11n_ modes inside the cylindrical resonant cavity, a rectangular waveguide is used at the bottom of the structure to filter out most of the noise waves. Adjusting the length of the cylindrical resonant cavity and the position size of the coupling hole achieves the *TE*_11n_ multi-mode resonance test environment in the cylindrical resonant cavity. Based on the analysis of the field distribution in the cylindrical resonant cavity and the *TE*_11n_ resonant cavity test principle, the relationship between the bottom diameter *D* of the cylindrical resonant cavity [29,30,31,32,33] and the number of resonant modes inside the resonant cavity at different frequencies can be plotted according to Equation (1). In the case that the bottom diameter is determined, it is easy to see that the selection range of the resonant cavity’s length varies with the range of the resonant peak through the figure. This brings great convenience to the subsequent rational design of the cylindrical resonant cavity.

(1) $$f_{0}=\frac{c}{2\pi }k=\frac{c}{2\pi }\sqrt{{\left(\frac{2u_{mn}}{D}\right)}^{2}+{\left(\frac{p\pi }{L}\right)}^{2}}$$

where *k* is the wave number, *c* is the speed of light, *D* is the bottom diameter of the cylindrical resonant cavity, *L* is the cylindrical height, and *u*_mn_ is the nth root of the mth order Bessel derivative function corresponding to the cutoff wavelength of the excitation mode number inside the cylindrical resonant cavity. The cutoff wavelength is affected by the perturbation of the coupling hole.

**Figure 1:**
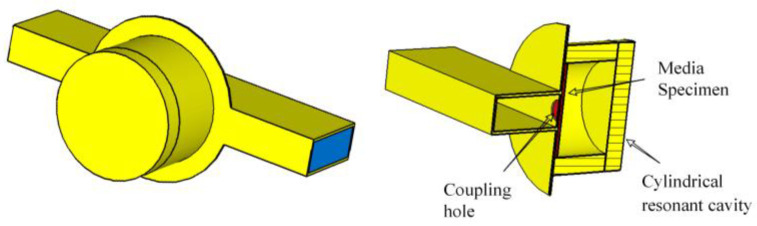
Cylindrical resonant cavity simulation model.

At the same time, the cutoff wavelength is also affected by the sample inputted. Meanwhile, to avoid the influence of other mode numbers and to allow the loss peak of the *TE*_11n_ mode to appear in the range required inside the resonant cavity, rational design of the cavity length *L* of the cylindrical resonant cavity is needed to achieve the role of purifying the mode in the cavity. After the dimensions of the cylindrical resonant cavity are determined, simulation software CST studio suite 2015 is used to model the resonant cavity, so samples put inside the resonant cavity for simulation can be tested in actual measurements. The model created by CST is shown in Figure 1. The resonant cavity consists of two parts. One is the cylindrical resonant cavity above and the other is the rectangular waveguide in the lower half. Inside the cylindrical resonant cavity, the circular waveguide mode *TE*_111_ as the lowest oscillation mode number has a field structure similar to that of the rectangular waveguide mode *TE*_011_ and can be easily obtained through the rectangular waveguide mode transition. The cylindrical resonant cavity has been well-designed with a reasonable cavity length *L* to produce *TE*_111_ one-cavity multimode electromagnetic field distribution with good single-mode. Meanwhile, the rectangular waveguide is used to connect with the external vector network tester and coupled with the cylindrical resonant cavity through a small coupling hole.

**Figure 2:**
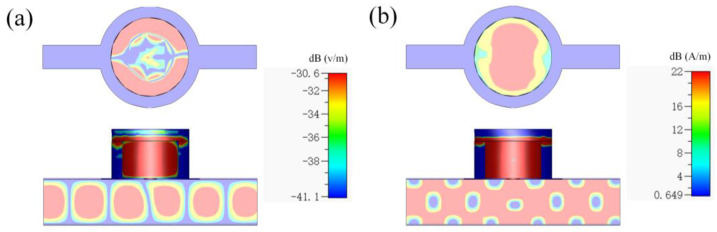
(**a**) Electric field distribution of the excited *TE*_111_ mode; (**b**) magnetic field distribution of the excited *TE*_111_ mode.

The sample for testing is placed inside the cylindrical resonant cavity covering the coupling hole, and the bottom diameter of the sample is 29 mm (*D* = 29 mm) as well as the cylindrical resonant cavity. The field distribution of the cavity resonant peak frequency point is monitored, as shown in Figure 2, and the resonant peak of the *TE*_111_ mode number as the dominant mode does appear in the cylindrical resonant cavity. Figure 2a,b shows the top and side profiles of the electric and magnetic field distributions, respectively. The number of modes inside the rectangular waveguide and the number of principal modes excited inside the cylindrical resonant cavity can be clearly seen. The colored bars on the right side indicate the intensities of the waves in different parts of the cylindrical resonant cavity and inside the rectangular resonant cavity.

It can be clearly seen that the absorption in the coupling hole region is obviously different from the absorption intensity in other regions. Because the *TE*_111_ mode inside the cylindrical resonant cavity is obtained from the *TE*_011_ mode inside the rectangular waveguide through the transition of the coupling hole, the size of the coupling hole directly affects the electromagnetic field boundary conditions inside the cylindrical resonant cavity.

In the simulation, resonant frequency point, half power point frequency difference (HPPFD), and other subsequent calculations of the sample in different permittivity and loss tangents can be obtained by setting the value of the sample’s thickness, permittivity, and loss tangent. These data were then substituted into the equations, and the preliminary calculation results were obtained by Mathematica. Based on the comparison of the simulation results and the calculation results, as well as the discovery of regularities from the plotted graphs, the correction direction of the formula was guided. Finally, the optimized correction coefficient values were obtained to form a complete test system. The reliability and feasibility of the system were further verified by testing actual samples with a vector network tester.

## 3. Results and Discussion

**Figure 3:**
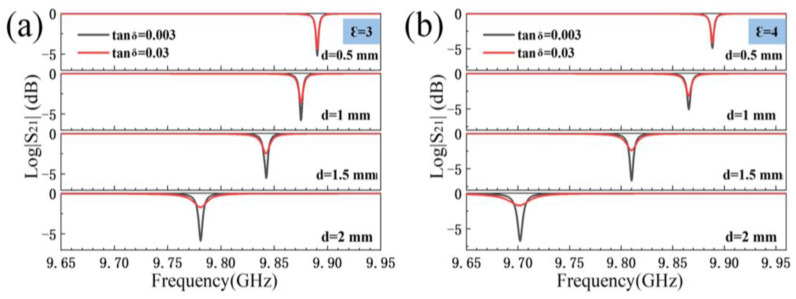
(**a**) Resonant peak variations due to different sample thicknesses when Ꜫ = 3; (**b**) resonant peak variations due to different sample thicknesses when Ꜫ = 4 (Tan δ represents the value of loss tangent).

To measure the permittivity of the low-loss material, it is necessary to find the variation inside the resonant cavity by different thicknesses, permittivities, and different loss tangents of the sample. The data are obtained and plotted by changing the three parameters of the sample through the settings window of the CST simulation software. Figure 3a,b corresponds to the cases when the permittivity of the samples is equal to 3 or 4, respectively. The change of the resonant peak inside the resonant cavity shows an obvious regularity when changing the sample thickness to 0.5 mm, 1 mm, 1.5 mm, or 2 mm in the two cases. When the permittivity is constant, the resonant peak inside the resonant cavity moves toward low frequency point with an increase of the sample thickness. It is obvious that the effect of permittivity on the movement of the resonant peak in the resonant cavity is greater than the thickness of the sample within a certain range.

**Figure 4:**
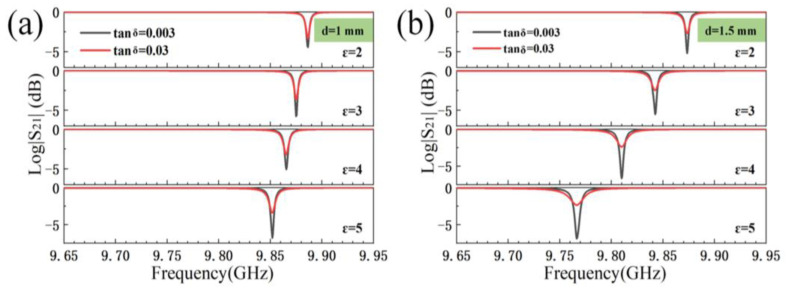
(**a**) Resonant peak variations due to different permittivities when d = 1 mm; (**b**) resonant peak variations due to different permittivities when d = 2 mm (Tan δ represents the value of the sample loss tangent).

In order to investigate in depth the effect of permittivity and sample thickness on the resonance peak, cases with the same sample thickness but different permittivity are studied. Figure 4a,b is the case of changing the permittivity values of the sample when the sample thickness is equal to 1 mm or 1.5 mm, respectively. It is obvious that when the sample thickness is constant, increasing the permittivity of the sample will cause the resonant frequency point of the resonant peak inside the resonant cavity to move toward lower frequency point. The resonant frequency point shows a linear relationship in absolute value with increasing permittivity for the sample with same thinkness.

As shown in Figure 3 and Figure 4, the change of loss tangent, permittivity, and sample thickness lead to the change of the resonant peak in the resonant cavity. It can be concluded that both the sample thickness and the permittivity variation will cause the resonant peak in the resonant cavity to move to lower frequency point. Moreover, the change of the loss tangent of the sample will affect the HPPFD of the resonant peak. It also can be seen that the larger the loss tangent of the sample, the larger the value of the HPPFD. In the meantime, the minimum absorption value of the resonant peak will shifted up or down. Furthermore, this has a guiding role in the direction of the correction of permittivity and the loss tangent equations later.

According to the theory of permittivity inside the cylindrical cavity [33], it can be known that the solution of the resonant cavity interior beyond Equation (2) is the permittivity of the sample. The present work refers to the principles and the equations in it. The model is not built in the same way as in the reference.

(2) $$\frac{\tan\left[d\sqrt{{\left(\frac{2\pi f}{c}\right)}^{2}Ꜫ-{\left(\frac{2u_{11}}{D}\right)}^{2}}\right]}{\sqrt{{\left(\frac{2\pi f}{c}\right)}^{2}Ꜫ-{\left(\frac{2u_{11}}{D}\right)}^{2}}}+\frac{\tan\left[\left(L-d\right)\sqrt{{\left(\frac{2\pi f}{c}\right)}^{2}-{\left(\frac{2u_{11}}{D}\right)}^{2}}\right]}{\sqrt{{\left(\frac{2\pi f}{c}\right)}^{2}-{\left(\frac{2u_{11}}{D}\right)}^{2}}}=0$$

where *d* denotes the thickness of the sample, *c* is the speed of light, *L* is the length of the cylindrical resonant cavity, *f* is the resonant peak frequency point when the sample is placed into the resonant cavity, *D* is the diameter of the bottom of the cylindrical resonant cavity, *u*_11_ is the first zero value of the first-order Bessel function, and *Ꜫ* and d in Equation (2) are the sample permittivity and thickness. Finally, the permittivity of the sample can be obtained by solving the transcendental equation, which is also the parameter that needs to be subsequently corrected.

**Figure 5:**
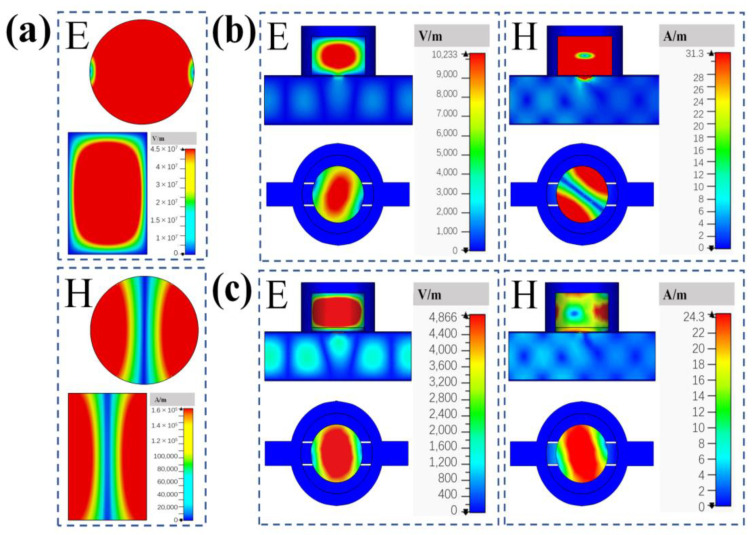
Electric and magnetic field distribution of cylindrical resonant cavities (**a**) without coupling hole and sample; (**b**) with coupling hole but without sample; (**c**) with coupling hole and sample.

As shown in Figure 5, the presence of the coupling hole and the dielectric material will change the boundary conditions of the electromagnetic field inside the cylindrical resonant cavity. As such, the cutoff wave number is no longer equal to the root of the Bessel derivative function divided by the radius of the cylindrical resonant cavity bottom.

Due to the distortion of the electromagnetic field distribution inside the cylindrical resonator, when calculating the permittivity with Equation (2), *u*_11_ = 1.841 cannot be used to calculate the permittivities and loss tangents of the samples inside the TE11 field that is distorted. As such, *u*_11_ = 1.841 needs to be corrected. Separately, Equation (2) is inaccurate for the calculation of permittivity, and the reasons include: (1) the influence of the coupling hole on the test; (2) the influence of the size of the sample on the internal electromagnetic field; and (3) the influence of the permittivity of the material itself on the measurement results. Combining the above considerations and influencing factors, it still returns to the original root of the problem, which is that the coupling hole and sample thickness will influence the cut-off frequency and mode number inside the cylindrical resonant cavity. The correction of *u*_11_ for the calculation the permittivity should take both into accoun. *u*_11_ is the first zero of the first-order Bessel derivative function, which is the corresponding characteristic root of Maxwell’s equations according to the electromagnetic field distribution law of the *TE*_111_ mode number. The thickness of the dielectric material as well as the permittivity will cause the distortion of the electromagnetic field distribution of *TE*_111_ inside the resonant cavity, which will affect the value of *u*_11_. Therefore, a correction of *u*_11_ is necessary.

**Figure 6:**
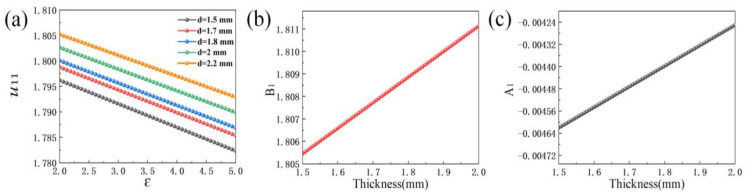
(**a**) Variation of *u*_11_ with Ꜫ at different sample thicknesses; (**b**) variation of correction factor *B*_1_ with sample thickness; (**c**) variation of correction factor *A*_1_ with sample thickness.

To explore the relationship between *u*_11_ and the permittivity and thickness of the sample, the relationship between the three quantities is verified in Figure 6a. In general, if the electromagnetic field distribution and the boundary conditions are normal inside the cylindrical resonant cavity, the reference value corresponding to the solution for each resonant mode number is the root of the Bessel derivative function. When the boundary conditions of the electromagnetic field change, the reference value cannot be used for calculation. Therefore, in order to obtain the correct value, the cutoff wave number needs to be corrected. It obvious that the slope of the variation curve of *u*_11_ with permittivity tends to be the same when the thickness of the sample changes from 1.5 mm to 2.2 mm, while the intercept and the thickness of the sample are also found to have a certain relationship through the analysis of each straight line. To simplify the relationship and to reduce the difficulty of the subsequent calculation of the loss tangent, it is worthwhile to write *u*_11_ in the form of *u*_11_(*Ꜫ*), where the permittivity *Ꜫ* is the independent variable and the thickness of the sample *d* is used as the correction parameter.; Then, the relationship between *u*_11_ and the two can be related. The effect of the loss tangent is not ignored in this work, *u*_11_ is virtually unaffected by the loss tangent, and the simplification of the equation is to reduce the complexity of calculating *u*_11_, thus saving time significantly. Equation (3) is to obtain the corrected value of *u*_11_ in Equation (2), and Equation (2) can be used to calculate the permittivity using the value of the resonant frequency point inside the resonant cavity without using the value of the HPPFD. As shown in Figure 3, changing the permittivity of the sample will directly lead to a shift of the resonant frequency point inside the resonant cavity, while changing the loss tangent will not lead to a shift. So, when correcting the permittivity, the effect of the value of the loss tangent value is almost negligible.

It may be useful to write the general expressions for *u*_11_ and sample thickness *d* and permittivity *Ꜫ* as Equation (3).

(3) $$u_{11}\left(\;Ꜫ\right)=A_{1}Ꜫ+B_{1}$$

Upon derivation and calculation, it was found that *u*_11_ is indeed represented by Equation (3), where *u*_11_ and permittivity are clearly linear, in line with the simplified equation. The correction factors are the slope of the line and the intercept value, which are only related to the thickness of the sample. *A*_1_ and *B*_1_ are functions of the sample thickness, respectively. *A*_1_ = *C*_1_d + *C*_2_, *B*_1_ = *C*_3_d + *C*_4_. After calculation, it is found that *C*_1_ = 0.74 × 10^−3^, *C*_2_ = −5.73 × 10^−3^, *C*_3_ = 0.0114, and *C*_4_ = 1.7884. The *A*_1_ and *B*_1_ of the *u*_11_(*Ꜫ*) straight line are plotted in Figure 6b,c.

In Figure 6, although the variation of *A*_1_ and *B*_1_ with different sample thicknesses is slow, it cannot be ignored because of the sensitivity of the measurement system, where slight changes in the correction factor and the sample thickness can have a dramatic effect on the final permittivity calculation.

**Figure 7:**
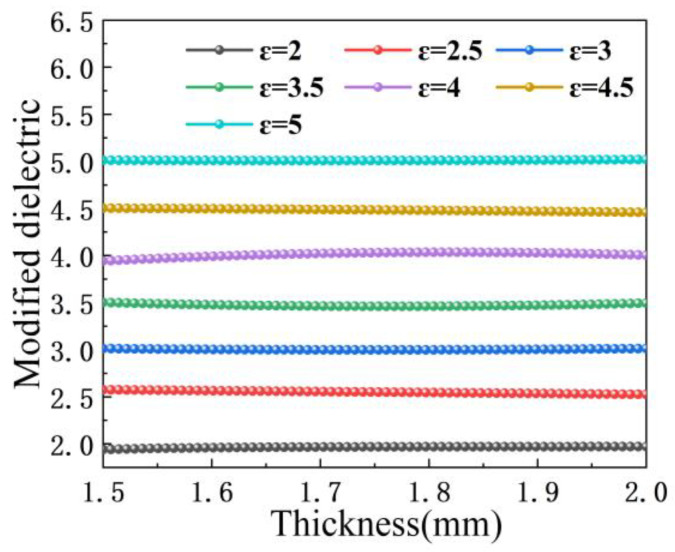
Comparison of actual and corrected permittivity at different sample thicknesses.

To verify the feasibility of the final correction results, low-loss dielectric samples were placed inside the cylindrical resonant cavity for permittivity measurements and the data obtained from the CST were substituted into the correction equation. Because the permittivity value of the sample is known, it is easy to obtain a comparison between the actual permittivity of the sample and the corrected permittivity shown in Figure 7. The different color lines correspond to the corrected values of the permittivity at different sample thicknesses, and the annotations of each line correspond to the actual permittivity value of the sample. The final correction results fluctuate around the actual permittivity value but are basically a constant straight line with very little deviation from the overall view, which indicates that the designed cylindrical resonant cavity can characterize the well-followed permittivity of the samples with a simple calculation.

In summary, the permittivity of the sample calculated by considering the sample thickness perturbation is more accurate, and the equation containing the correction factor is simpler than calculating the sample permittivity by using the value of the Bessel derivative function corresponding to *TE*_111_.

This is followed by a correction for the loss tangent, which is calculated in the cylindrical resonant cavity medium theory [34,35,36] by Equation (4). According to the equation, the loss tangent calculation of the sample is obtained by multiplying two parts. One part is related to the permittivity of the sample, while the correction of the permittivity part has been given previously. At the same time, the second part is related to the quality factor inside the resonant cavity; to be precise, it is related to the difference between the inverse of the no-load quality factor and the on-load quality factors. The subsequent equation shows that the calculation of the quality factor and the size of the sample are related.

(4) $$tan\delta =\left(1+\frac{u}{pv{Ꜫ}^{\prime }}\right)[\frac{1}{Q_{0s}}-\frac{1}{Q_{00}^{\prime }}]$$

(5) $$\frac{1}{Q_{00}^{\prime }}=\frac{1}{Q_{00}}{\left(\frac{f_{00}}{f_{0s}}\right)}^{\frac{5}{2}}\frac{\left[{\left(\frac{2X_{1n}}{D}\right)}^{2}\left(pv+u\right)+D\left(p\beta_{Ꜫ}^{2}+\beta_{0}^{2}\right)\right]}{\left(pv{Ꜫ}^{\prime }\right)[{\left(\frac{2X_{1n}}{D}\right)}^{2}\left(1-\frac{D}{L}\right)+{\left(\frac{2\pi f_{00}}{c}\right)}^{2}\frac{D}{L}]}$$

(6) $$p=\left[\frac{sin[\beta_{0}\left(L-d\right)]}{sin(\beta_{Ꜫ}d)}\right]{}^{2}$$

(7) $$u=2\left(L-d\right)-\frac{\sin\left[2\beta_{0}\left(L-d\right)\right]}{\beta_{0}}$$

(8) $$v=2d-\frac{\sin\left(2\beta_{Ꜫ}d\right)}{\beta_{Ꜫ}}$$

where *Ꜫ* is the permittivity of the sample, *X*_1n_ is the correction equation *u*_11_(*Ꜫ*), and *β*_Ꜫ_ and *β*_0_ are the phase constants in the resonant cavity with the sample and without the sample, respectively. *f*_00_ is the frequency point value of the resonant peak in the unloaded cylindrical resonant cavity, and *f*_0s_ is the frequency point value of the resonant peak in the loaded cylindrical resonant cavity after putting the sample. *Q*_00_ is the cylindrical resonant cavity intrinsic quality factor and *Q*’_0s_ is the no-load quality factor of a hypothetical test chamber with an ideal consumption-free sample. *Q*_0s_ is the unloaded quality factor of the test chamber after the actual sample is placed in the resonant cavity. To better demonstrate Equation (4), the parameters among the equations are expressed as Equations (5)–(8), and they are the relevant equations calculated by the resonant cavity perturbation method. Therefore, the loss tangent correction should not only involve the permittivity of the sample, but also consider the actual size of the sample.

Similarly, Equation (4) cannot describe the value of the loss tangent of the sample very accurately for the following reasons: (1) the conductivity of the inner surface of the resonant cavity and the number of modes inside the resonant cavity lead to a low quality factor of the cylindrical resonant cavity; and (2) the correction found that the permittivity of the sample also has a certain influence on the correction of the loss tangent. After the sample is placed inside the cylindrical resonant cavity, it will not only affect the cavity length (air part) but also the perturbation of the coupling hole to the cut-off wavelength (*u*_11_) inside the resonant cavity. The influence of the cavity length will inevitably change the value of the conductivity on the inner surface of the cylindrical cavity. Therefore, in addition to the original permittivity correction with the *u*_11_, it is necessary to consider the influence of the thickness of the sample itself on the loss tangent calculation.

**Figure 8:**
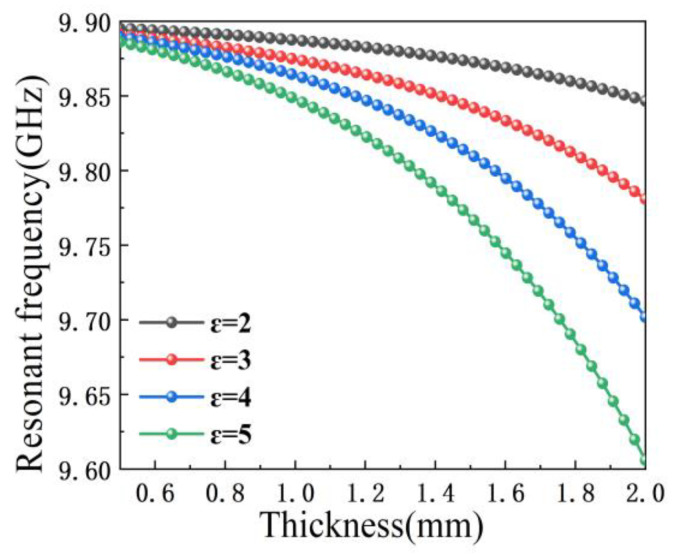
Dependence of resonant frequency on sample thickness with different permittivity.

Equation (4) is well understood and the data coming out of the simulation software are combined to guide the direction of the correction of the loss tangent. Figure 8 shows the change of resonant frequency points with the change of the testes sample thickness under different conditions. The rule that can be obviously seen is that whether the sample thickness becomes larger or the permittivity becomes larger, this will cause the resonant frequency point to move to the lower frequency point. However, when the permittivity is relatively low, it seems that the effect of the sample thickness is higher than the permittivity, and after a certain equilibrium point, the relationship between the two effects will be swapped. This regularity is extremely similar to the ones found in Figure 4 and Figure 5. The combination of these figures shows that to obtain the loss tangent value of the sample, it must be correlated with both permittivity and sample thickness.

**Figure 9:**
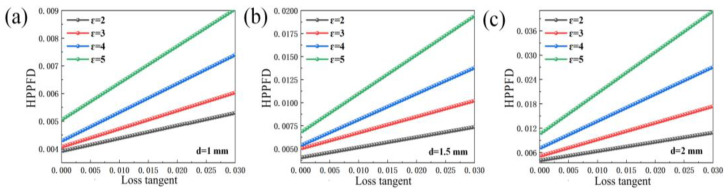
Dependence of the HPPFD on loss tangent with different permittivity when (**a**) d = 1 mm; (**b**) d = 1.5 mm; and (**c**) d = 2 mm.

Figure 9 is the HPPFD changing with the loss tangent when the sample thickness is 1 mm, 1.5 mm, or 2 mm, respectively. It is clear that when the sample thickness is the same, if the permittivity of the sample becomes larger, the HPPFD will also become larger as the loss tangent becomes larger; if the permittivity is set aside, when the permittivity of the sample is the same, if the thickness of the sample becomes larger, the loss tangent will cause the HPPFD to become larger. If the permittivity is set aside, when the permittivity of the sample is the same, if the thickness of the sample becomes larger, the loss tangent will cause the HPPFD to become larger. Furthermore, within a certain range, these lines will not intersect. Nevertheless, the relationship between the loss tangent and the permittivity and sample thickness shown in the figure is linear. This indicates that the equation has the possibility of correction.

To reduce the computational complexity of the loss correction equation, the corrected loss tangent is calculated after substituting the previously corrected permittivity into the equation for sample thicknesses d = 1.5 mm, 1.7 mm, and 2 mm. This is to ensure the closed-loop nature of the calculation. The actual dielectric constant and loss tangent of the sample will be combined with thickness-dependent coefficients to finally obtain the correct loss tangent. The relational expression is written in the form of Equation (9).

(9) $$tan\delta =A_{2}Ꜫ+B_{2}tan\delta^{\prime }+C$$

The simulation-obtained data are substituted into Equation (9), and then the corresponding correction coefficients *A*_2_, *B*_2_, and *C* for the three sample thicknesses can be obtained and plotted in Table 1. From Table 1, the three coefficients are different at different sample thicknesses. *A*_2_ varies with sample thickness in a large and then small way, and *B*_2_ varies with sample thickness in a small and then large way. On the contrary, the coefficient C varies with sample thickness in a monotonic way.

**Figure 10:**
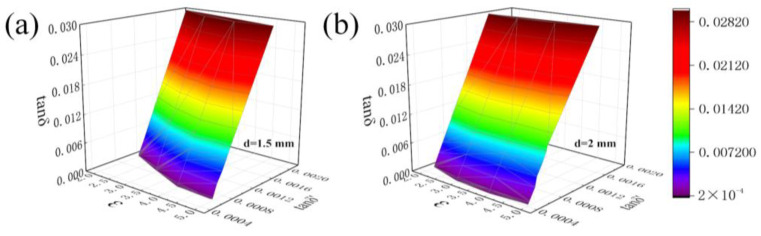
(**a**) Correction results for a sample with a thickness of 1.5 mm; (**b**) correction results for a sample with a thickness of 2 mm.

Figure 10 shows the three-dimensional diagrams of the tested sample thicknesses of 1.5 mm and 2 mm, respectively. It is obvious that when the sample permittivity is used as the *X*-axis and the loss tangent before correction is used as the *Y*-axis, the final loss tangent appears on the *Z*-axis. It can be seen that although the thickness of the samples is different, the actual loss tangent of the sample can be accurately calculated by the correction factors (*A*_2_, *B*_2_, and *C*), the actual permittivity, and the thickness of the sample. This further proves that the loss tangent of the sample is related to the permittivity and the thickness of the sample.

**Figure 11:**
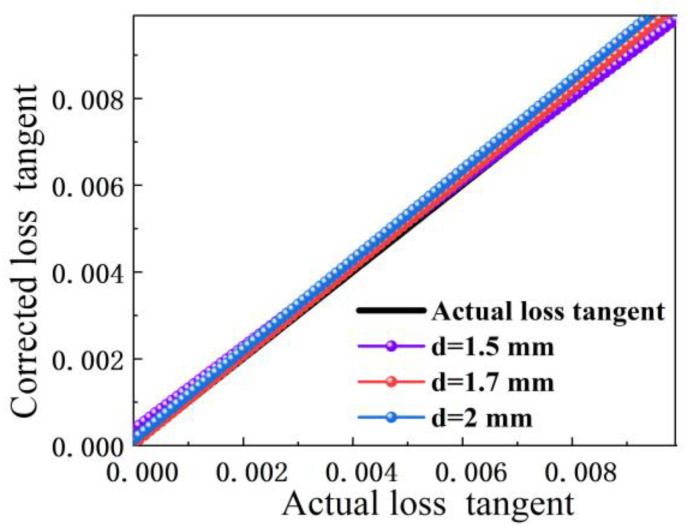
Comparison of actual loss tangent and corrected loss tangent of the sample.

To see the difference between the corrections and the actual value more obviously, Figure 11 is presented for the comparison of the actual loss tangent and the corrected loss angle tangent of the sample. The horizontal coordinate X represents the actual loss tangent value of the sample, and the vertical coordinate Y represents the actual loss tangent or corrected loss tangent value of the sample, which should have an obvious linear relationship between the two. It is obvious that the different sample thicknesses (three colored lines) and the actual loss tangent (black line) almost coincide; the black lines are the corrected loss tangent values for sample thicknesses of 1.5 mm, 1.7 mm, and 2 mm, respectively, showing that the final results fluctuate around the actual values. This further illustrates that the corrected results are very close to the actual results. For samples with different thicknesses of 3.00 mm or 1.96 mm, the initial goal of this work was to unify all thicknesses into a single equation. However, it was found that the corrected permittivity could be unified into a formula, while the corrected equation for the loss tangent could only be unified for a specific thickness.

In summary, after considering the influence of the perturbation of the coupling holes and the inner wall of the cavity on the loss tangent calculation results, different formulas for calculating the loss tangent were derived, and the accuracy of the loss tangent calculation is also greatly improved. This work is aimed at low permittivity and low-loss tangent materials, which have a permittivity of 2–5 and a loss tangent of 0.0003–0.03. Most low-loss dielectric materials are in this range. A comparison of dielectric properties measurement by different resonant cavities is shown in Table 2. The test method in this work has the advantages of high measurement accuracy and small sample diameter. In particular, it is not necessary to prepare a test sample with a specific thickness according to permittivity.

To further verify the practicality of this work, some samples were tested using a vector network tester and a cylindrical resonant cavity. Considering that the thickness of the sample and the coupling hole on the resonant cavity will affect the resonant cavity peak, the different thicknesses of samples will cause the resonant peak frequency point to move to the left at different distances. Therefore, when designing the cavity length of the cylindrical resonant cavity, we tried to meet the test conditions, i.e., and keep the resonant peak position inside the resonant cavity at no load as close as possible to the right. The permittivity and loss tangent magnitudes of different samples tested with the vector network tester are listed in Table 3.

**Figure 12:**
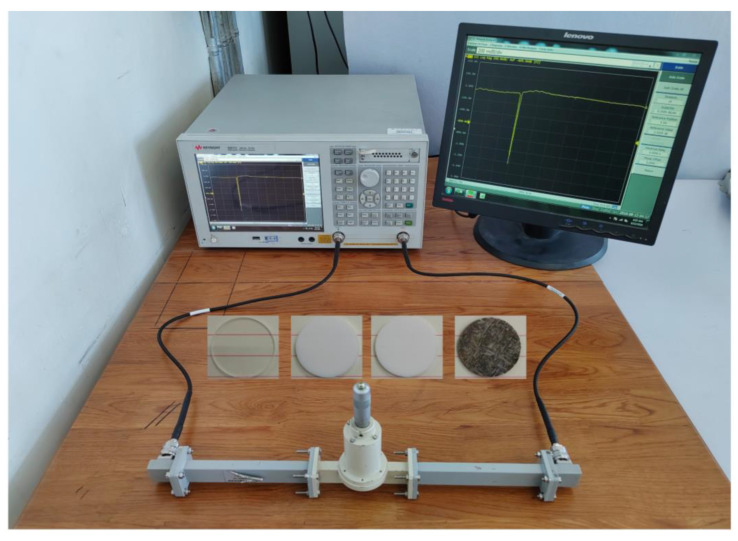
Photo of vector network tester and different samples.

Shown in Figure 12 are the systematic tests for the quartz, PTFE, and silicon oxide ceramics, respectively. As can be seen from Table 3, although the calculated permittivity and loss tangent are not the same for different sample thicknesses of PEFT, the difference is not much, and they are both above an order of magnitude. Of course, if the same sample is tested, different densities will also affect the accuracy of the test, but the impact is not significant.

## 4. Conclusions

A resonant cavity detecting the permittivity and loss tangent of low-loss materials was designed, and the traditional calculation formula was modified based on the electromagnetic field distribution of the *TE*_111_ mode. The new method proposed can accurately measure the complex permittivity of low-loss materials with smaller sizes. Retrieval of permittivity is carried out by introducing the perturbation of the cutoff wavenumber caused by the coupling hole and the sample size. The precise loss tangent of the low-loss material is obtained by determining its dependence on permittivity and sample thickness. The measurements of standard samples verified that this method has high accuracy in characterizing the electromagnetic properties of low-loss materials.

## Figures and Tables

**Table 1:** Correction factors at different sample thicknesses.

Thicknesses (mm)	A_2_	B_2_	C
d = 1.5	−0.0436	27.5348	−0.0436
d = 1.7	−0.0079	26.0791	−0.0189
d = 2	−0.0091	27.0574	−0.0044

**Table 2:** Comparison of dielectric properties measurement by different resonant cavities.

Resonant Cavity Types	Resonant Mode	Permittivity Test Range	Loss Tangent Test Range	Diameter or Length and Width (mm)	No Need to Prepare a Test Sample of a Specific Thickness according to Permittivity	References
Cylindrical	*TE*_111_	2.92–3.22	0.03–0.048	D = 49	No	[34]
Rectangle	*TE*_111_	10	0.002	l = 11.5, w = 7	Yes	[35]
Rectangle	*TE*_011_	1–20	-	l = 23, w = 15	Yes	[36]
Cylindrical	*TM*_0n0_	8.3–8.36	0.1	D = 40	No	[37]
Cylindrical	*TM*_010_	2–3.5	0–0.09	D = 37	No	[38]
Rectangle	*TE*_0mn_	21.2	-	l = 71.86, w = 138.52	No	[39]
High Q cavity	*TE*_01n_	38–88	0.0002–0.001	D = 50	No	[40]
Cylindrical	*TE*_111_	2–5	0.0003–0.03	D = 29	Yes	This work

**Table 3:** Measurement results of different standard samples.

Sample Name	Thickness (mm)	Density (g/cm^3^)	Resonant Frequency Point (GHz)	HPPFD (GHz)	Permittivity	Loss Tangent
Quartz	1.50	2.20	10.0437	0.0059	4.1	4.7 × 10^−4^
PTFE 1	2.00	2.28	10.0581	0.0037	2.1	6.9 × 10^−4^
PTFE 2	3.00	2.24	9.9391	0.0056	2.0	4.5 × 10^−4^
Silicon oxide ceramics	1.96	1.57	10.0126	0.0276	3.0	3.7 × 10^−3^

## Data Availability

The data that support the findings of this study are available from the corresponding author upon reasonable request.
